# A Novel Web-Based Experiential Learning Platform for Medical Students (Learning Moment): Qualitative Study

**DOI:** 10.2196/10657

**Published:** 2018-10-17

**Authors:** Alexander Y Sheng, Andrew Chu, Dea Biancarelli, Mari-Lynn Drainoni, Ryan Sullivan, Jeffrey I Schneider

**Affiliations:** 1 Department of Emergency Medicine Boston Medical Center Boston, MA United States; 2 Boston University School of Medicine Boston, MA United States; 3 Department of Health Law, Policy and Management Boston University School of Public Health Boston, MA United States; 4 Evans Center for Implementation and Improvement Sciences Boston University School of Medicine Boston, MA United States; 5 Section of Infectious Diseases, Department of Medicine Boston University School of Medicine Boston, MA United States; 6 Center for Healthcare Organization and Implementation Research Edith Nourse Rogers Memorial Veterans Hospital Bedford, MA United States; 7 Lawrence General Hospital Lawrence, MA United States

**Keywords:** experiential learning, reflection, shared learning

## Abstract

**Background:**

Experiential learning plays a critical role in learner development. Kolb’s 4-part experiential learning model consists of concrete experience, reflective observation, abstract conceptualization, and active experimentation in a recurring cycle. Most clinical environments provide opportunities for experiences and active experimentation but rarely offer structured means for reflection and abstract conceptualization that are crucial for learners to learn through experience. We created Learning Moment, a novel Web-based educational tool that integrates principles of asynchronous learning and learning portfolios to fulfill the reflection and abstract conceptualization aspects of Kolb’s learning cycle in the modern clinical learning environment. Medical students log concise clinical “pearls” in the form of “learning moments” for reflection, review, and sharing with peers in a community of practice.

**Objective:**

We sought to evaluate learners’ experiences with Learning Moment via a qualitative study.

**Methods:**

We employed purposive sampling to recruit medical students who used Learning Moment during their rotation. We conducted 13 semistructured interviews (10 individual interviews and one 3-person group interview) between January and March 2017 using an ethnographic approach and utilized a general inductive method to analyze and code for potential themes.

**Results:**

A total of 13 students (five in their third year of medical school and eight in their fourth year) voluntarily participated in our qualitative interviews. Five of the 13 (38%) students intended to pursue emergency medicine as their chosen field of specialty. The median number of “learning moments” logged by these students is 6. From our analysis, three key themes emerged relating to the perceived impact of Learning Moment on student learning: (1) logging “learning moments” enhanced memorization, (2) improved learning through reflection, and (3) sharing of knowledge and experiences in a community of practice.

**Conclusions:**

Learning Moment was successfully implemented into the educational infrastructure in our department. Students identified three mechanisms by which the application optimizes experiential learning, including enabling the logging of “learning moments” to promote memorization, encouraging reflection to facilitate learning, and fostering the sharing of knowledge and experiences within a community of practice. The Learning Moment concept is potentially scalable to other departments, disciplines, and institutions as we seek to optimize experiential learning ecosystems for all trainees.

## Introduction

Experiential learning, which incorporates both reflection and practice, is critical for successful knowledge acquisition, educational growth, and the development of new skills and behaviors [1,2]. In Kolb’s 4-part model of the experiential learning cycle, (1) concrete experience forms the foundation for learning, (2) reflection subsequently makes sense of the experience alone or in a group setting, (3) abstract conceptualization uses reasoning and knowledge to grasp the situation and problem, and (4) active experimentation puts theories to the test, leading to additional experiences [2,3].

With widespread acceptance of experiential learning as a crucial component of medical education [4], prior research examined the application of Kolb’s work in relation to curriculum design for continuing medical education [5]. However, there is a lack of literature exploring ways to integrate and optimize experiential learning in the modern clinical learning environment. In addition, there is increasing recognition of the importance of reflective practice in the development of medical professionals [6]. Although incorporated into medical training through the use of simulation debriefing sessions, written reflections in narrative medicine, and verbal reflections in problem-based and case-based learning, few published approaches exist to encourage reflection during day-to-day clinical practice [6-8]. While the clinical learning environment provides opportunities for experiences and active experimentation aspects of Kolb’s learning model, it often lacks structured opportunities for reflective observation and abstract conceptualization. In response to these challenges, educators and learners seek alternative frameworks and mechanisms to support and foster continuous experience, learning, and reflection that can co-exist and thrive within today’s changing health care landscape.

One such alternative is the use of asynchronous learning as a learner-centered method of teaching that uses online learning resources to overcome time and space constraints in order to facilitate information sharing and interaction among learners [9]. Importantly, asynchronous learning has been demonstrated to be a valued and effective method of learning [10,11]. Another method to encourage learning through experience is the use of learning portfolios, which is predicated on three fundamental components: reflection, documentation, and collaboration [12]. As described by Stanton, the use of learning portfolios stimulates additional (and self-perpetuating) learning as reflection on experiences leads to the recognition of new learning goals, which in turn renews the learning cycle [1].

We developed and implemented an innovative educational tool, Learning Moment [13], integrating principles of asynchronous learning and learning portfolios, to fulfill the reflection and abstract conceptualization aspects of Kolb’s experiential learning cycle often missing in the clinical learning environment. We sought to evaluate learners’ experience with this novel educational instrument via a qualitative study.

## Methods

### Educational Tool Design

**Figure 1:**
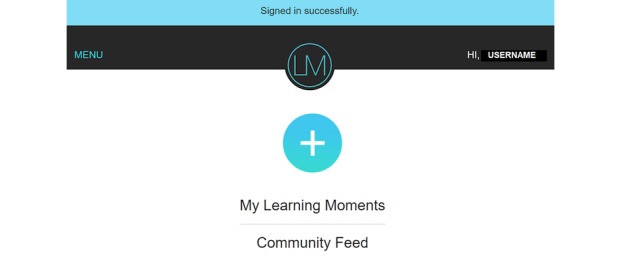
Learning Moment interface.

Learning Moment is a Web-based application through which learners can document and share personal learning experiences that occur in the course of patient care (Figure 1). Learners are able to conveniently and easily record “learning moments” (defined as learner-identified learning experiences), highlight the take-away “learning pearls,” and share them with peers. Through this process, learners have the opportunity to incorporate key components of Kolb’s experiential learning cycle, reflective observation and abstract conceptualization in particular, that are frequently absent in the bustle and chaos often present in today’s clinical learning environment.

**Figure 2:**
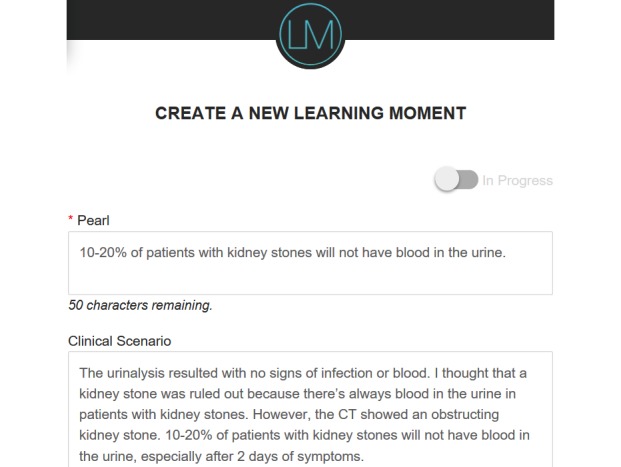
Logging learning experiences in Learning Moment. CT: Computerized Tomography.

**Figure 3:**
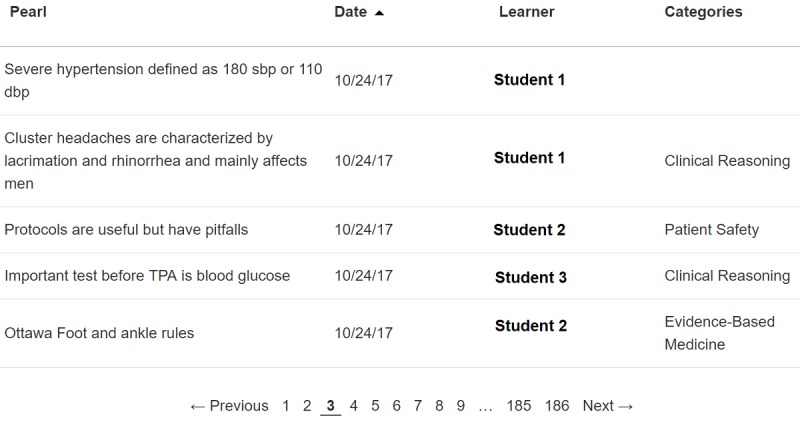
Learning Moment Community Feed. TPA:Tissue Plasminogen Activator.

We developed the initial build of the Learning Moment electronic platform with two specific goals in mind:

1. To provide learners with an electronic “note-taking” tool to log their own learning experiences (Figure 2) while building and contributing to their own digital personalized learning portfolio. In doing so, we endeavored to provide learners with a physical and mental space to synthesize experiences into coherent thoughts, which enhance understanding and retention through self-reflection and abstract conceptualization [14].
2. To create a searchable and shareable repository of useful, practical, high-yield educational content that benefits peers and colleagues through vicarious learning in the form of a “Community Feed” (Figure 3) [15]. Our intention was to build and support a community of practice, both live and virtual, to facilitate knowledge sharing [16,17].

### Implementation

The complete description of the Learning Moment implementation process is described elsewhere [13]. In brief, we implemented Learning Moment in August 2016 at a busy (annual volume in excess of 130,000 visits), urban, tertiary care emergency department that hosts an emergency medicine residency and robust third- and fourth-year medical student clerkships. We introduced the Learning Moment platform to all rotating medical students during their orientation to the clerkship. We encouraged them to use Learning Moment on a voluntary basis to log self-selected learning experiences in the form of a “learning moment” and to view fellow students’ “learning moments” through a “Community Feed” (Figure 3). Students can access the Learning Moment website on computer workstations at work, at home, or on their mobile phones.

A link to the Learning Moment website was made accessible directly from the electronic medical record system to promote ease of access. Experienced clinical faculty facilitated monthly in-person “Learning Moment Reflection” small group discussions with medical students as they reflected on and expounded on their own and peer “learning moments” with faculty guidance. We designed these discussions to complement the virtual features of Learning Moment to further encourage and fulfill the reflection and abstract conceptualization components of experiential learning.

A total of 323 “learning moments” were logged between August, 22, 2016 to Feb. 12, 2017, over the course of six 1-month-long clerkship rotations. Over three-quarters (42/53, 79.2%) of medical students who completed their emergency medicine clerkship rotation logged at least one “learning moment” with a median of six “learning moments” logged. The demographics of the medical student user cohort and frequency of logging “learning moments” are described previously [13]. We recently surpassed 1000 “learning moments” logged 16 months after implementation, demonstrating successful implementation and robust sustainability.

### Study Design and Recruitment

We conducted a qualitative analysis of users’ experience of Learning Moment as a learning tool for undergraduate medical education. We employed purposive sampling to recruit medical students who used Learning Moment during their rotation. We sent email invitations with subsequent reminders to all students who rotated in our emergency medicine clerkship from August 2016 to February 2017 to participate in qualitative interviews, regardless of the extent to which they used the Learning Moment platform. Our interviews focused primarily on how medical students used Learning Moment throughout their emergency medicine rotation and how it impacted their learning. We coded the data inductively using the principles of grounded theory to generate a unified, theoretical explanation on how Learning Moment impacted the learning experience of medical students. Our Institutional Review Board approved our study as exempt.

### Data Collection Procedures

We conducted 13 semistructured interviews, including 10 individual interviews and one 3-person group interview, between January and March 2017. We conducted seven interviews in person and six by telephone due to difficulty arranging face-to-face meetings. In-person interviews were conducted in medical school classrooms and departmental conference rooms. We conducted interviews until we reached thematic saturation as the last several interviews yielded no additional patterns or themes. A single researcher and coauthor (AC) conducted and audiotaped all interviews using the same interview guide (see Multimedia Appendix 1). Individual interviews lasted between 5 and 20 minutes with a mean and median of 15 minutes and 16 minutes respectively. The 3-person group interview was 26 minutes in duration.

### Data Analysis

After each interview was completed, the researcher and coauthor (AC) who conducted the interviews transcribed the audio recording verbatim. We reviewed all transcribed interviews to ensure accuracy. For analysis, we employed standard qualitative research methods using the principles of grounded theory [18,19]. Two coauthors (AC and DB) trained in qualitative research methods inductively analyzed the transcripts, generating common themes found in all interviews. We initially reviewed the text line-by-line and coded them to characterize comments and passages and subsequently grouped them into conceptual categories to form an initial codebook. The same coauthors then applied the initial codebook to transcripts, refining and finalizing the codebook for a “better fit” for the data. We applied the new version of the codebook by both team members to all the transcripts using qualitative software package NVivo (QRS International). After several rounds of refining and finalizing the coding scheme, we identified overall themes related to the impact of Learning Moment on student learning and appropriately grouped them into thematic categories.

## Results

### Description of the Study Sample

In total, 13 students (five in their third year of medical school and eight in their fourth year) of the 53 who rotated with us during the study period voluntarily participated in our qualitative interviews. Five of the 13 (38%) students intended to pursue emergency medicine as their chosen field of specialty. Detailed demographics of participants are shown in Table 1.

The number of “learning moments” logged during their month-long emergency medicine rotation by each of the 13 students who participated in our qualitative interviews are shown in Table 2. The median number of “learning moments” logged by these students is 6.

### Overview of Qualitative Themes

The interview data provided a deeper understanding of learner views and experiences using Learning Moment. Three key themes relating to the impact of Learning Moment on student learning emerged from our analysis: (1) learners expressed that the act of logging of “learning moments” enhanced memorization, (2) learners attributed self-perceived improvement in learning to reflection, and (3) learners appreciated sharing knowledge and experiences in a community of practice.

#### Theme 1: Learners Expressed that Logging “Learning Moments” Enhanced Memorization

Learners believed that the physical act of logging a “learning moment” facilitated memorization. Learners appreciated the platform to record information in an easily digestible format thus enabling future review and stimulating knowledge retention.

**Table 1:** Demographics of participating medical students (N=13).

Characteristics		Participants, n (%)
**Year**		
	Third year of medical school	5 (38)
	Fourth year of medical school	8 (62)
**Gender**		
	Female	11 (85)
	Male	2 (15)
**Field of interest**		
	Emergency Medicine	5 (38)
	Other or unsure	8 (62)

**Table 2:** Number of “learning moments” logged during their month-long emergency medicine rotation by each student who participated in our qualitative interviews.

Student	“Learning moments” logged, n
1	3
2	13
3	6
4	2
5	14
6	8
7	9
8	2
9	4
10	6
11	5
12	9
13	22

If I don’t write them down or type them out, then I don’t remember. I like to have them logged in somewhere so, one day, when I review them, I’m like “Oh yeah, I remember learning about that,” and that solidifies what I learned on my rotation.

Student 3

The thing I took from it the most was the reinforcement of my own learning. Usually you hear clinical pearls from attendings or residents, and you can think about it for that moment. But then you might forget it later on...actually being able to type it down in this database really reinforces that information that you just learned. And then, I think because of that, I was able to retain this information much better.

Student 6

Not only was the act of typing out “learning moments” viewed as helpful for memorization, but students also commented that recording these “pearls” and thus building their learning portfolios was an effective method to capture, easily organize, and review this information. Students noted that when documenting new learning “pearls” they often looked back and reviewed previously recorded “pearls,” thus refreshing their memory of those previous learning experiences:

You also have the ones you wrote down in the past, so it all kind of jogs your memory. I think it’s a memory tool that lets you quantify some kind of learning that you had every day for every shift.

Student 3

Later on, it helped me remind me of things I had learned, and that I might have forgotten I have, so it was nice to have that refresher. Because the ones I input are the ones that I remember best.

Student 4

#### Theme 2: Learners Attributed Self-Perceived Improvement in Learning to Reflection

Learners reported that the often chaotic and frenetic pace of a busy emergency department made it difficult to synthesize and reflect on learning experiences. Learners articulated that Learning Moment provided an intentional and deliberate moment to pause and reflect on the learning that occurred that day and allowed them to think about how to apply it in the future. Through this process, learners recounted more opportunities to reinforce the learning that does occur.

So, I think that being in the emergency room is a very unique setting. So, when you’re in the emergency department as a student, things are very fast-paced...I think in those really quick moments, there are moments of learning and teaching that’s happening that might not otherwise be apparent. So even though you're working in a fast-paced learning environment...the program really helped me to reflect in that way to synthesize...after a really busy 8-hour shift.

Student 12

The one good thing that it did was that it forced me to stop after every shift...to think about what they actually learned. Because it’s so easy to pop in for eight hours, go in, do your thing, and then leave...not really reflect back on what had happened that day. Because the emergency room is pretty busy, so being able to stop and think about what had happened...that retrospective approach was one of the benefits that LM [Learning Moment] offered.

Student 5

Most useful was the reflecting. You know, because in a shift, you see a whole bunch of patients. So, reflecting on: was there anything new or different about one of those patients who came in, so that we can think about that patient in the future when other patients come in.

Student 8

So, I think that the main purpose of it is to give you a chance to step back from the action-packed environment of the emergency department to give you a pointed purpose and opportunity to think beyond what’s going on right now.

Student 9

#### Theme 3: Learners Appreciated Sharing Knowledge and Experiences in a Community of Practice

Learners believed that the sharing of learning “pearls” facilitated knowledge transfer within a community of practice. Learned enjoyed reviewing the Learning Moment “Community Feed” to learn from the “pearls” and experiences of their peers. Learners appreciated that every “learning moment” recorded was highly applicable in the clinical setting and might not be taught in traditional sources, such as textbooks. Learners frequently referenced that sharing their learning experiences benefited everyone within the community of practice and that Learning Moment encouraged “an environment of teaching” [Student 12].

But also, be able to tap into clinical pearls that other people in the department are gathering. Sort of like collect all that information in one space so you can have like a high yield bank of pearls and things that’ll help you on the floor...And so it is really nice that I could type in a keyword and get all of these clinical pearls, both my own and other people’s. And I liked the fact that we could learn from other people’s pearls.

Student 7

And skimming through other people’s “learning moments” was also useful and interesting. One of the benefits of using LM.

Student 6

I think the purpose of the LM was to encourage an environment of teaching. So not only was it to have students and residents reflect on things that they learned during their shift...also to encourage attendings and more senior providers to teach more and provide those learning moments for students on shift. I think that was encouraged, so that aspect of the program was pretty cool.

Student 12

Learner experiences using Learning Moment were impacted by the level of involvement by others from the ground up. While the current iteration of Learning Moment is focused on medical students in a single emergency medicine clerkship rotation, increasing participation and support from residents and faculty as well as adapting Learning Moment to other rotations are potential future directions as suggested by our learners.

I think the thing with LM is that you need a lot of buy-in for it to be good...if I were using that on every single rotation, or if it were in my residency and everyone in my residency was using it...I would totally use it, because I think it’s a good tool. If everybody’s using it or is using it consistently throughout the year, I would totally use it.

Student 7

I think if it was part of the curriculum where I was, it would be useful. I don’t think if I was just doing it my own thing that I would use it. If it wasn’t a part of my residency, I don’t know that I would use it.

Student 2

Incorporating into the culture of the residency. I remember when I was rotating through, somebody brought up LM during our weekly conference and was like, “Oh look at all these learning moments,” and clearly, someone was looking at it, and someone shared different learning moments, and that would be encouraging more people to participate. Whereas if it was something that wasn’t utilized, if I was the only one doing it, then it would be a little harder to incorporate into my daily tasks.

Student 1

## Discussion

### Principal Findings

While there is literature supporting the need for educators to design continuing medical education curriculums that transition learners through each stage in sequence of the Kolb experiential learning cycle in order to promote the application of all learning styles [5], there is no such structured approach to support learners in the same way in the clinical environment. Without such infrastructure, learners may fail to assimilate and transform experiences into knowledge [2,4]. Learners are assumed to learn during clinical work through experience in a productive manner despite the lack of structure or framework that fosters learning and reflection [20]. As a result, some have advocated for the creation and incorporation of curricular spaces within the medical curriculum and clinical settings in order to provide opportunities for learners to incorporate experiences into their professional identity through reflection [21]. For example, Shaughnessy and Duggan introduced reflective exercises in the form of Web-based “clinical blogs” into their family medicine residency curriculum. While perceived as valuable for self-development, the participating residents expressed unease in setting aside dedicated time for reflection amid the professional duties and time pressures they face on a daily basis [22]. In most clinical settings, particularly within a busy emergency department, it may be even more difficult to devote valuable time protected from clinical duties solely for the purposes of reflection. Learning portfolios have been increasingly used in various health professions including medicine, nursing, and dentistry to promote learner metacognition and even faculty development [23-29]. However, the vast majority of portfolios used in undergraduate medical education featured reflective writing focusing on ethical and professional issues, as opposed to day-to-day clinical learning [23]. One e-Portfolio was used to define learning activities of final year medical students during the emergency medicine clerkship by recording patient demographics, chief complaints, and procedures. However, consideration of learning pearls or sharing of knowledge and experiences were not mentioned [30].

To explicitly address these gaps, we sought to integrate the concepts of asynchronous learning and learning portfolios, while leveraging technology, to fulfill aspects of Kolb’s learning cycle that are often absent in the clinical learning environment, specifically reflection and abstract conceptualization. According to Kolb, learning is an active, collaborative, and interactive process “whereby knowledge is created through the transformation of experience” [2]. Our results suggest that Learning Moment facilitates the transformation of clinical experiences into existing cognitive frameworks. Reflection can occur *during* (reflection in action) or *after* the experience (reflection on action), and both are critically important for the development of reflective physicians [14]. Learners can leverage the Learning Moment platform for reflection on action, by logging “learning moments” asynchronously (online) or through in-person “Learning Moment Reflection” discussion groups. Additionally, Learning Moment also provides a potential means by which learners can reflect in action or in the context of an active educational experience that allows for opportunity to do so.

Furthermore, our data suggest that the Learning Moment platform encourages group learning through sharing of knowledge and experiences within a community of practice. Learning Moment creates a shareable repository of knowledge that benefits the community of practice through vicarious learning [15]. In such an environment, knowledge acquisition can occur not only via first-hand experiences but also indirectly through the experiences of others. There is an increasing awareness and understanding within higher education of students’ ability to learn from each other’s experiences [31-34]. But vicarious learning is not well described in the medical education literature despite its widespread use (eg, through story telling in social interactions, structured discussions like morbidity and mortality conferences). Learning Moment’s “Community Feed” feature, which fosters the sharing of knowledge and experiences through vicarious learning, was identified as a strength by users.

Terms like learning management system, course management system, and virtual learning environment have been used somewhat interchangeably to describe integrated suites of tools used in e-learning such as Blackboard [35], New Innovations [36] and E-Value [37]. They are effective and widely used tools that support the administration, logistics, communication, and book-keeping aspects of educational courses and programs [38]. Learning Moment on the other hand, through its innovative approach to learner documentation and sharing of learning experiences, can genuinely be considered a virtual learning environment in the true sense of the term because it not only supports the educational process but also generates valuable educational content and maintains a virtual environment in which to maximize experiential learning.

### Limitations

Our study has several important limitations. The sample size was small with only 13 voluntary interview participants. Logistic restrictions from rigorous schedules of medical students, many of whom were from out of state, doing rotations away, or traveling while interviewing for residency positions during the study period significantly limited our efforts to conduct more interviews. As a result, we performed one 3-person group interview in order to accommodate participant schedules. Despite a relatively small sample size, we reached thematic saturation. Learners who self-selected to participate in the study may have strong positive or negative views towards Learning Moment, thus subjecting our results to participation bias. Although in our recruiting email describing the voluntary nature of participation, we stressed that participation would not affect their grade or ranking for residency application in any way, participants may have been motivated to report positive experiences with Learning Moment, thus biasing our results. In addition, our study did not include a control arm. Since we sought to evaluate learners’ experiences with Learning Moment, interviewing learners who did not use Learning Moment was not part of our initial study design. However, exploring reasons behind why some learners did not use Learning Moment despite its availability during the rotation would have been a worthy venture. Last, our results reflect learner perceived impact of Learning Moment on their learning. We cannot comment on specific learning outcomes as we did not measure knowledge acquisition, retention, or change in practice, specifically. Nevertheless, our study was the first of its kind to explore learner experiences regarding their use of our innovative experiential learning platform.

### Conclusion

We successfully implemented Learning Moment into the educational infrastructure of our department. Student users identified three mechanisms by which the application was perceived to optimize experiential learning, including enabling the logging of “learning moments” to promote memorization and retention, encouraging reflection to facilitate learning, and fostering sharing of knowledge within a community of practice. While currently institution-based, further research addressing refinements to and enhancements of the tool, as well as potential adoption of the Learning Moment model to new learning environments, potentially as an open-access platform, is warranted as we seek to optimize experiential learning ecosystems for all trainees.

## Appendix

Multimedia Appendix 1 Interview guide.

## Abbreviations

LM: Learning Moment
