# Supplementary material for: A Novel Web-Based Experiential Learning Platform for Medical Students (Learning Moment): Qualitative Study
Source: JMIR Med Educ. 2018 Oct 17;4(2):e10657. doi: 10.2196/10657 (PMC6231881; doi:10.2196/10657)
Supplement: Multimedia Appendix 1 [file mededu_v4i2e10657_app1.pdf]

Interview Guide
Questions for the participating medical student:

1. What do you think is the purpose of *Learning Moment*?
2. How often did you use *Learning Moment* as a learning tool?
3. How did you use the *Learning Moment* as an educational tool? How did it help you or not?
4. What did you like about *Learning Moment*? And not like?
5. What parts of the *Learning Moment* were most useful to you, and why?
6. What parts of the *Learning Moment* were least useful to you, and why?
7. Would you continue to use *Learning Moment* after your emergency medicine rotation and during residency?
    a. If so, how would you use it?
    b. If not, why not?
8. What functions and capabilities should the website have in order for it to be useful as a learning tool during your rotation and afterwards?
9. If it were to have these functions, how would you envision yourself using it?
10. Is the information filtered in a useful way? How could it be improved?
11. How could we change the design and layout of our website so that you could interact with it in a way that's useful to you?
